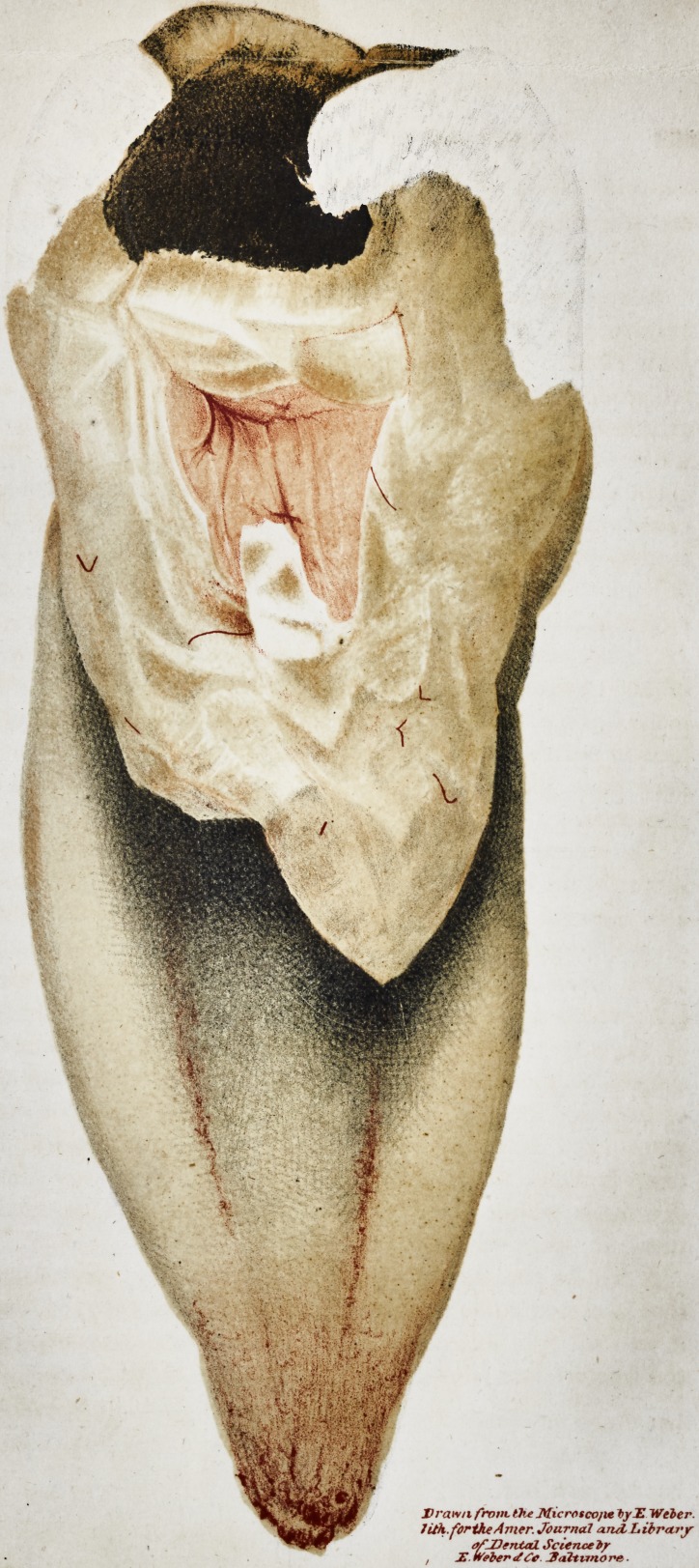# On the Vascularity of Dental Bone

**Published:** 1842-03

**Authors:** Chapin A. Harris


### ARTICLE V.

### *On the Vascularity of Dental Bone.* By CHAPIN A. HARRIS, M. D., D. D. S.

ALTHOUGH the doctrine of the nonvascularity of the teeth, as advanced by the celebrated English anatomist and physiologist, John Hunter, has been shown, over and over again, by facts which one would suppose none could doubt, to be erroneous, the existence of vessels in dental bone, has not hitherto, that the writer of this, is aware of, been actually demonstrated. By the aid of a microscope, he, however, has been fortunate enough to discover vessels charged with red blood in the substance of human tooth-bone, in two instances. The first time he done it, was a little more than twelve months ago, and it was in a thin section which he had cut from a molar tooth of a very young person. In this a vessel was seen charged with red blood, and it was exhibited to several medical gentlemen, and to the class of the Baltimore College of Dental Surgery. This section of tooth is at this time in possession of the writer, and may be seen by any one, who may have the curiosity to examine it, by calling on him at his residence.

The second time he had the good fortune to make this discovery, it was in the half of an inferior molaris, taken from the mouth of a boy, eleven years of age, and of which, an exact representation of a microscopic view of it, is here annexed. The tooth had ached violently for several days previously to its extraction, and from which circumstance, he was induced to believe, that the vessels of the pulp were highly injected, and to satisfy himself upon the subject, he, soon after its removal, split it open with a strong pair of excising forceps. As was anticipated the vessels of the pulp were filled with red blood, and on examining the half of the tooth in which this had remained, through a microscope, a number of vessels within the very substance of the bone, charged with this fluid, were also distinctly seen.

It will be perceived by an examination of the annexed drawing that a considerable portion of the crown had decayed, and that it was not split exactly in the centre between the roots, but that the fracture had passed partly through one of the fangs.

Drawn from the Microscope by E. Weber.

lith. for the Amer. Journal and Library of Dental Science by

E. Weber & Co. Baltimore.

The fractured surface, the decayed cavity in the crown of the tooth, the pulp and vessels within the bone charged with red blood are all so distinctly seen, that no other explanation is deemed necessary.

## Figures and Tables

**Figure f1:**